# Mulberry polyphenols ameliorate atherogenic migration and proliferation by degradation of K-Ras and downregulation of its signals in vascular smooth muscle cell

**DOI:** 10.7150/ijms.76006

**Published:** 2022-09-06

**Authors:** Ching-Pei Chen, Yi-Liang Wu, Kuei-Chuan Chan, Hsieh-Hsun Ho, Chau-Jong Wang, Li-Sung Hsu

**Affiliations:** 1 Division of Cardiovascular Surgery, Surgical Department, Chung Shan Medical University Hospital, Taichung 402, Taiwan; 2 Department of Surgery, School of Medicine, Chung-Shan Medical University, Taichung 402, Taiwan; 3 School of Medicine, Chung Shan Medical University, Taichung 402, Taiwan; 4 Department of Internal Medicine, Chung Shan Medical University Hospital, Taichung, 402, Taiwan; 5 Institute of Biochemistry and Biotechnology, Chung Shan Medical University, Taichung, 402, Taiwan; 6 Department of Health Diet and Industry Management, Chung Shan Medical University, Taichung 402, Taiwan; 7 Department of Medical Research, Chung Shan Medical University Hospital, Taichung, 402, Taiwan; 8 Institute of Medicine, Chung Shan Medical University, Taichung, 402, Taiwan; 9 Department of Medical Research, Chung Shan Medical University Hospital, Taichung 402, Taiwan

**Keywords:** mulberry polyphenol extracts (MPE), vascular smooth muscle cells, migration, proliferation, K-Ras

## Abstract

Extra-proliferation and increased migration of vascular smooth cells con-tribute to the formation of atherosclerosis. Ras small G proteins play a critical role in the prolif-eration and migration of a wide range of cells. Mulberry, an economic fruit in Asia, exhibits anti-inflammation, anti-migration, and anti-oxidant properties. The mechanisms of action of mulberry extracts on K-Ras small G protein-induced proliferation and migration of vascular smooth muscle cell have not been extensively investigated. In this study, we explored the effects of mulberry polyphenol extracts (MPE) on the proliferation and migration of K-Ras-overexpressing A7r5 smooth muscle cells. The overexpression of K-Ras enhanced the ex-pression and activity of matrix metalloproteinase (MMP)-2, promoted vascular endothelial growth factor (VEGF) production, and eventually triggered the migration of A7r5 cells. Treatment with MPE attenuated K-Ras-induced phenomenon. In addition, MPE blocked K-Ras-induced actin fibril stress. MPE dose-dependently diminished K-Ras-induced Rho A, Rac1, CDC42, and phosphorylated focal adhesion kinase (FAK) expression. MPE elevated Rho B ex-pression. Phosphorylated AKT and glycogen synthase kinase (GSK) induced by K-Ras were also repressed by MPE treatment. MPE enhanced the interaction of IκB with NFκB. MPE restored the G0/G1 population and p21 and p27 expressions, which were repressed by K-Ras. Finally, MPE triggered the degradation of K-Ras by ubiquitination. MPE inhibited the migration and proliferation of vascular smooth cell through K-Ras-induced pathways and eventually pre-vented atherosclerosis.

## Introduction

Atherosclerosis contributes to the development of cardiovascular diseases, including myocardial infarction and ischemic stroke; the high prevalence of atherosclerosis is a cause of mortality worldwide 1. Normally, vascular smooth muscles control vascular contraction, blood pressure, and extracellular matrix (ECM) generation 2. During atherosclerosis progression, the proliferation of vascular smooth muscle cells (VSMCs) is first increased; then, they migrate into the intima layer to differentiate into macrophage-like VSMCs. The macrophage-like VSMCs trigger extensive ECM formation and promote fibrous cap formation 2, 3. Agents that regulate the proliferation and migration of VSMC may benefit the treatment of atherosclerosis.

Ras small G proteins transduce signals from receptors to cytosolic downstream targets and play a critical role in multiple cellular functions, such as proliferation, migration, and differentiation. Many reports demonstrated that Ras family proteins are involved in atherosclerosis formation by provoking the migration and proliferation of vascular smooth muscle. Yang et al. demonstrated that oxidized low density lipoprotein (ox-LDL) promotes vascular smooth muscle proliferation through enhanced Ras/Raf/MEK/MAPK pathway 4. In addition, Ox-LDL promotes the GTP loading (up to 9-fold) of Ras and activates downstream signals that trigger the proliferation of aortic smooth muscle cells 5. Lactosylceramide enhances superoxide production by activating NADPH oxidase and then stimulates the proliferation of smooth muscle by promoting Ras-dependent pathway, eventually causing atherosclerosis 6. Researchers focused on preventing atherosclerosis by using agents that block the activation of Ras signals. High glucose medium damages vascular endothelial cells (VECs), which then release growth factors to stimulate the proliferation of vascular smooth cells, thereby facilitating atherosclerosis formation 7. Paeonol decreases VEC-induced growth factor level and inhibits Ras expression in response to high glucose treatment 7. Lin et al. demonstrated that TW-01, a piperazinedione-derived compound, significantly diminishes Ras/Raf/mitogen-activated protein kinase (MAPK) activities and then attenuates the proliferation of VSMC 8. Yu et al. showed that acarbose significantly inhibits Ras expression in a dose-dependent manner in A7r5 cells 9. Moreover, acarbose also reduces phosphoinositide-3-kinase (PI3K)/Akt signaling and diminishes the migration and proliferation of A7r5 cells that show forced expression of activated Ras 9.

Mulberry (*Morus spp*., Moraceae), is an economic plant that is widely cultivated in Asian countries, such as China, Korea, and Japan. It has been used as a folk medicine for a long time 10. Extracts from mulberry have pharmacological properties, including antioxidant, anti-inflammation, and anti-diabetes activities 10. The effects of mulberry on anti-atherosclerosis have received attention. Administration of 1% mulberry leaf powders obviously reduced the atherosclerotic lesions to 40% compared with a normal diet group in apolipoprotein E-deficient mice 11. Chan et al. showed that mulberry leaf polyphenols mitigated the proliferation of vascular smooth muscle by enhancing p53 activity and repressing cyclin-dependent protein kinase 2/4 activities 12. Oral administration of mulberry leaf extracts also decreased the fasting blood glucose and reversed endothelial dysfunction parameters, which then prevented atherosclerosis in diabetic rats 13. Mulberry leaf tea and it major ingredient deoxynojirimycin significantly repressed cerebrovascular and cardiovascular events and reduced carotid intima-media thickness in patients with coronary heart disease in a clinical trial 14. The polyphenol contents of mulberry are very similar to those of mulberry leaf, thereby suggesting that mulberry polyphenols (MPE) may also play a role in anti-atherosclerosis treatment 15. MPE significantly reduced migration and triggered apoptosis in smooth muscle cells 15, 16. However, the role of mulberry on K-Ras-induced smooth muscle migration and proliferation still remains unclear. In the present study, we investigated the effects of mulberry polyphenol extracts (MPE) on the proliferation and migration of K-Ras-overexpressing smooth muscle cells.

## Materials and methods

### Materials

All chemicals were obtained from Sigma-Aldrich Company (St. Louis, MO, USA). The antibody for p16 (Sc-1661), p21 (Sc-6246), p27 (Sc-1641), MMP-2 (sc-10736), NFκB (Sc-109), IκB (Sc-847), ubiquitin (Sc-8017), Ras (Sc-32), RhoA (Sc-418), RhoB (Sc-8048), CDC42 (Sc-87), VEGF (Sc-4570), and AKT (Sc-5298) were purchased from Santa Cruz Biotech (Santa Cruz, CA, USA). The antibodies for FAK (CST#3285), PI-3K (CST#4255), phosphorylated AKT (CST#9275,), phosphorylated FAk (CST#3281), and integrin β3 (CST#4702) were obtained from Cell Signaling Technology (Beverly, MA, USA). Anti-actin (A-5316,) was purchased from Sigma-Aldrich Company.

### Cell culture

The rat aorta smooth muscle A7r5 cells purchased from American Type Culture Collection (ATCC, Manassas, VA, USA) were kept in Dulbecco's modified Eagle's medium (DMEM) supplemented with 10% fetal bovine serum (FBS), 1% glutamine, and 1% penicillin-streptomycin. These cells were incubated at 37 °C in a humidified atmosphere of 5% CO2. The K-Ras transfection procedures were performed as reported previously 17.

### Preparation and characterization of MPE

The MPE was extracted as reported previously 12. Dried mulberry (100 mg) was dissolved in 500 ml methanol and heated at 50 °C for 3 h. The methanol solution was filtered and lyophilized under reduced pressure at room temperature. The powders were resuspended in 500 mL of 50 °C distilled water and extracted by 180 mL of ethyl acetate thrice. After lyophilization, the powders were dissolved in 300 ml water and store at -80 °C until use. The polyphenol contents were analyzed by high performance liquid chromatography (HPLC) as reported previously 15. The mobile phase contained two solvents: A, 2% acetic acid/water (2:98, v/v); and B, 0.5% acetic acid in water/acetonitrile (50:50, v/v). Phenolic acids were detected at 260 nm. Quantitative assessment of the percentage of polyphenols in MPEs relative to the standards was conducted. Results represent the average of three independent experiments. The polyphenol contents listed in Table 1 are similar to those presented in a previous report 15. The major compounds in MPE were epigallocatechin gallate, protocatedchuic acid, rutin, caffeic acid as listed in Table 1. Three g of MPE was obtained from 100 g mulberry, the recovery ratio was 3%.

### Cytotoxicity assay

The A7r5 cells were seeded into 24 well plates at a density of 3 × 10^4^, and then treated with indicated concentration of MPE for 24 and 48 h. The survival rate was determined by 3-(4,5dimethylthiazol-2-xl)-2,5 diphenyl-tetrazolium bromide (MTT) assay as previous report 16.

### Reverse-transcriptase-polymerase chain reaction (RT-PCR)

Total RNA from A7r5 cells with different treatments was extracted using TRIzol reagent according to the manufacturer's protocols. RNA (4 µg) was transcribed into first strand cDNA by Moloney Murine Leukemia Virus (M-MLV) reverse transcriptase and oligo-dT primer. PCR was performed using specific primers as listed in Table 2.

### Immunoprecipitation and Western blot analysis

For immunoprecipitation, 500 μg cell lysates were pre-cleaned with agarose-A beads and then incubated with IkB antibody plus agarose-A beads for overnight at 4 °C. The immuno-complexes were washed by centrifugation and subjected to Western blot analysis.

A7r5 cells were lysed in RIPA buffer containing proteinase inhibitors. Protein concentration was measured by Bio-Rad protein assay kit. Proteins (50 μg) were separated by sodium dodecyl sulfate polyacrylamide gel electrophoresis and transferred into polyvinylidene difluoride (PVDF) membrane. After blocking by phosphate buffered saline (PBS) containing 5% non-fat milk, the membrane was incubated with a specific first antibody. After washing with PBS plus 0.1% Tween-20, the membrane was reacted with horseradish peroxidase (HRP)-conjugated second antibody, and signals were detected by using an enhanced chemiluminescence kit.

### Gelatin zymography analysis

The A7r5 cells transfected with K-Ras were cultured in serum-free medium treated with or without MPE for 24 h. The conditional medium was collected, and gelatin zymography containing 0.1% gelatin was conducted to detect the activities of MMP-2 and MMP-9 as reported previously 15.

### F-actin staining

A7r5 cells were seeded at a density of 5 × 10^4^ in a 6-well plate and subjected to the indicated treatment. Cells were fixed with 4% formalin and permeated by 0.1% Triton-X 100. After washing with PBS, cells were incubated with 500 μg/ml phalloidin-TRITC for 30 min in the dark. After washing with PBS, the images were captured by confocal microscopy with 540 mm excitation wavelength and 570 mm emission wavelength. The nucleus was stained with DAPI staining solution.

### Migration assay

A7r5 cells subjected to the indicated treatments were plated in the upper chamber of the 48 well Boyden chamber at a density of 5 × 10^5^/mL. The lower chamber was filled with medium containing 10% FBS. The chamber was incubated at 37 °C for 24 h. The migrated cells were fixed with methanol for 10 min and then stained with Giemsa solution. Cells were captured in three random fields under 400 × magnification.

### Cell cycle analysis

The A7r5 cells transfected with K-Ras were treated with or without 1 mg/mL MPE for 24 h. The cells were collected, fixed with ice-cold 75% ethanol, and stored at -20 °C. The cells were stained with 50 μg/mL propidium iodine (PI) and 100 μg/mL RNase A and analyzed by flow cytometer (Becton Dickinson, CA, USA). The cell cycle distribution was measured by CellQuest Software (Becton Dickinson, CA, USA).

### Statistical analysis

Data were presented as means and standard deviation and were measured from at least three independent experiments. Paired t test was used to detect the difference between groups by SPSS software. A *p* value of < 0.05 was considered as significant difference.

## Results

### MPE downregulated K-Ras-induced MMP-9 and VEGF expression

**Figure 1:**
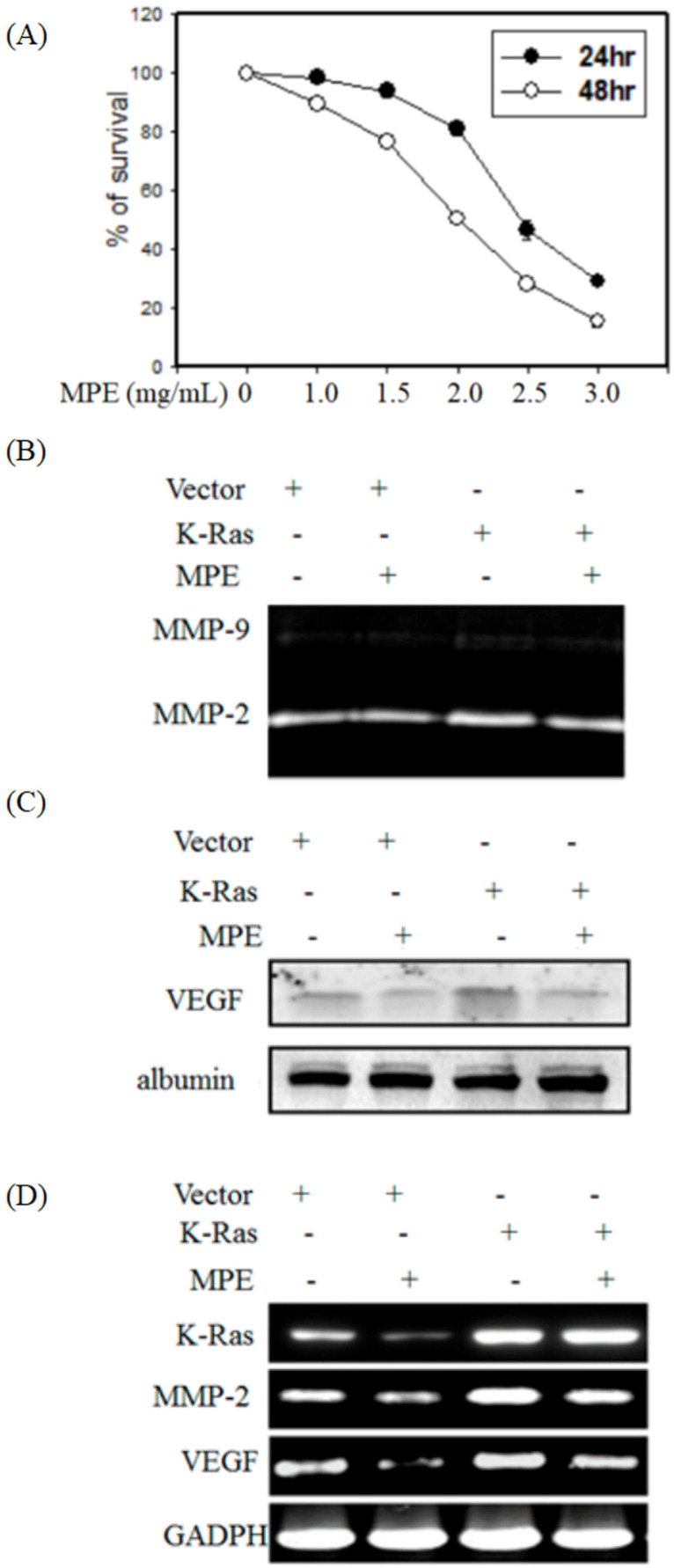
** MPE downregulated K-Ras-induced migration-related proteins' expression. (A)** A7r5 cells were treated with 0, 1.0, 1.5, 2.0, 2.5, and 3.0 mg/mL MPE for 24 or 48 h. The survival rate was determined by MTT assay. A7r5 cells with or without K-Ras overexpression after treatment with 0 or 0.5 mg/ml MPE for 24 h. **(B)** MMP-2 and -9 activities were assayed by gelatin zymography. **(C)** VEGF expression was analyzed by Western blot. **(D)** RT-PCR was performed to detect K-Ras, MMP-2, and VEGF expressions. Albumin and GAPDH were used as internal control.

First, to evaluate the cytotoxicity of MPE on smooth muscle cells, MTT assay was performed. The cell survival tare was decreased in s dose- and time-dependent in the presence of 1 to 3 mg/mL MPE (Fig. 1A). To avoid the cytotoxicity effects, we selected the concentration of 0.2 and 0.5 mg/mL MPE for further experiments.

To detect whether K-Ras and MPE regulated the activities of MMP-2 and -9, gelatin zymography was performed. MPE reduced MMP-2 activity in the absence or presence K-Ras expression (Fig. 1B). Western blot analysis showed that K-Ras elevated vascular endothelial growth factor (VEGF) expression, whereas MPE treatment reversed K-Ras-induced VEGF expression (Fig. 1C). RT-PCR results also demonstrated that MPE repressed K-Ras-induced MMP2 and VEGF expressions (Fig. 1D).

### MPE mitigated K-Ras-induced cell migration and F-actin structure

**Figure 2:**
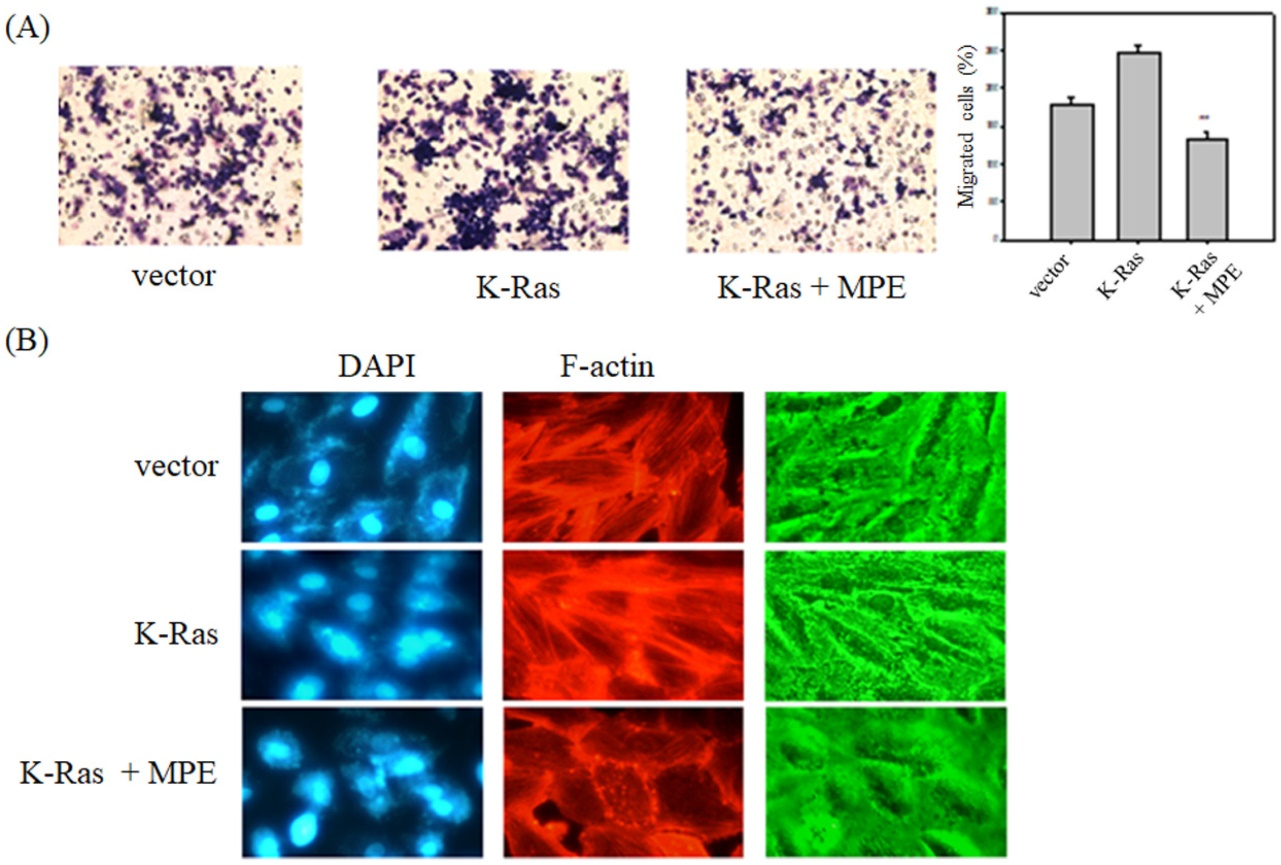
** MPE inhibited K-Ras-induced migration and F-actin stress formation.** A7r5 cells with or without K-Ras overexpression were treated with 0 or 0.5 mg/ml MPE for 24 h. **(A)** Migration was analyzed by Boyden chamber. Data represented means ± standard deviation. **: P < 0.01 compared with K-Ras overexpression group. **(B)** F-actin and nucleus were stained by phalloidin-TRITC and DAPI, respectively. The image was captured under confocal microscopy.

To determine the effects of MPE on K-Ras-induced migration of vascular smooth cells, A7r5 VSMCs with transient overexpression of K-Ras (A7r5-K-Ras) were used, and Boyden chamber analysis was performed in the presence or absence of MPE. As shown in Fig. 2A, K-Ras obviously triggered the migration of A7R5 cells, whereas MPE (0.2 mg/ml) significantly mitigated K-Ras-induced migration.

**Figure 3:**
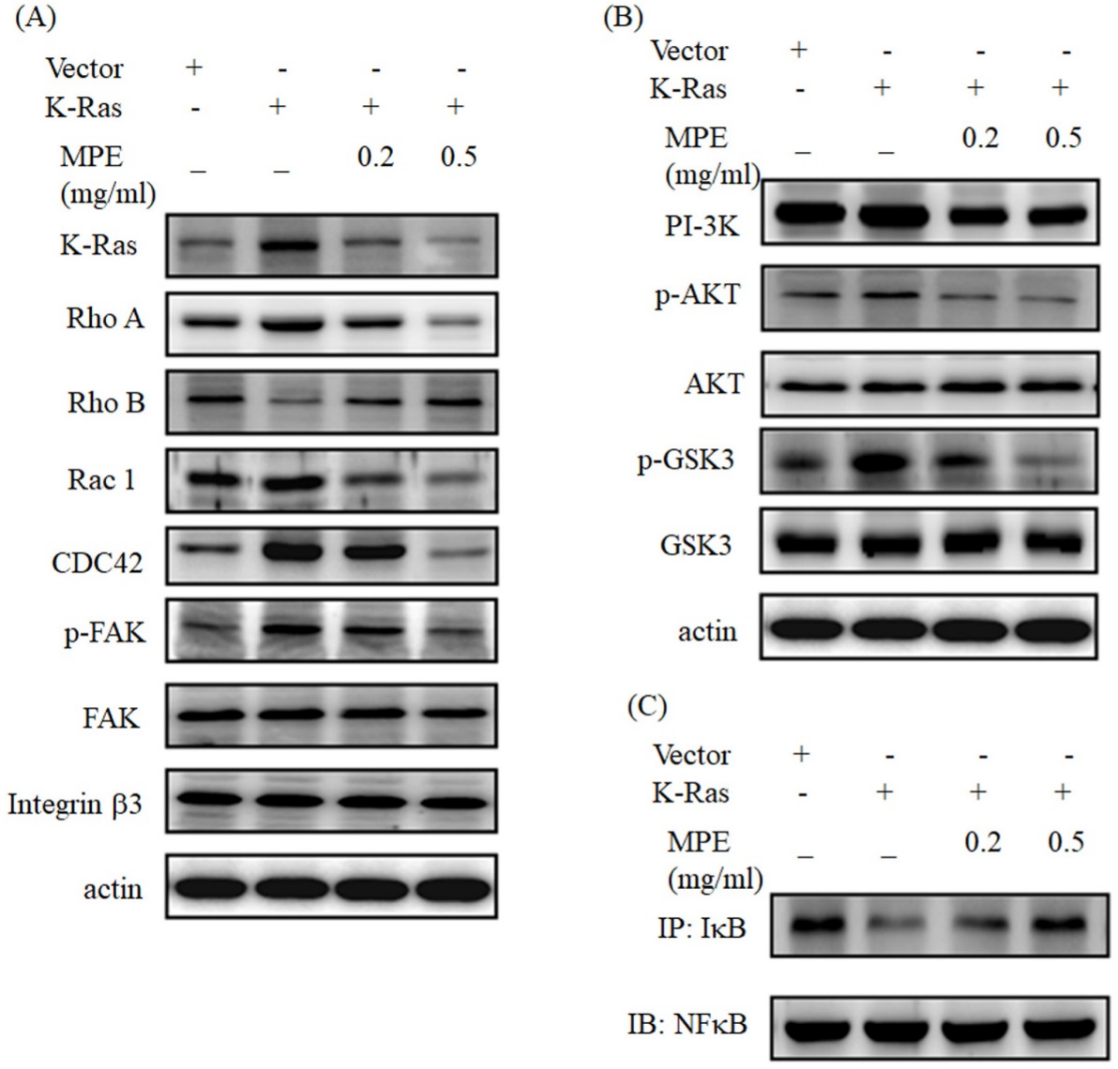
** MPE reversed the effect of K-Ras on migration-related protein expressions and NFkB activity. (A and B)** A7r5 cells with or without K-Ras overexpression treated with 0 or 0.5 mg/ml MPE for 24 h. Protein expression was detected by Western blot analysis using the indicated antibody. **(C)** The interaction of IkB and NFkB was assayed by immunoprecipitation using the anti-IkB antibody followed by Western blot analysis using the anti-NFkB antibody.

Western blot analysis showed that K-Ras obviously promoted Rho A, Rac 1, and CDC42 expressions. Phosphorylated focal adhesion kinase (FAK) was increased in K-Ras overexpression cells, but no alternation of FAK and integrin β3 was found. K-Ras expression suppressed and MPE recovered the Rho B expression (Fig. 3A). K-Ras triggered the expression of phosphoinositide 3-kinases (PI3K). In addition, K-Ras elevated phosphorylated AKT and glycogen synthase kinase (Fig 3B). MPE dose-dependently reversed the effects of K-Ras (Figs. 3A and 3B). K-Ras blocked the interaction of IkB with NFkB, whereas MPE recovered the interaction in a dose-dependent manner (Fig. 3C).

### MPE attenuated K-Ras-induced cell cycle progression

**Figure 4:**
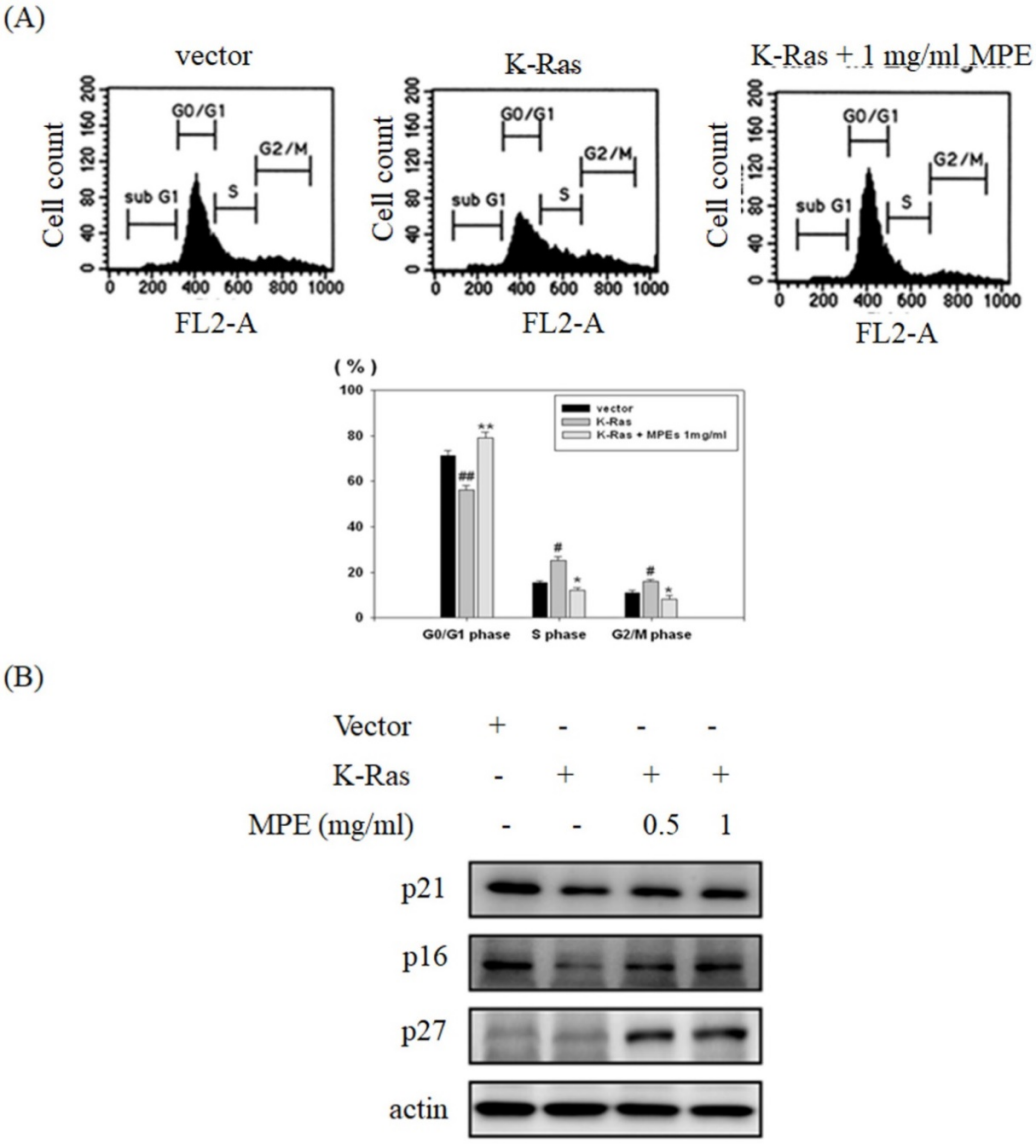
** MPE recovered K-Ras-induced cell cycle arrest and cell cycle inhibitor expression. (A)** The cell cycle populations of A7r5 cells transfected with vector plasmid, A7r5 cells with K-Ras overexpression, and A7r5 cells with K-Ras overexpression plus 1 mg/ml MPE treatment were measured by flow cytometry analysis. Data represented means ± standard deviation. # and ##: P < 0.05 and P <0.01 compared to vector alone cells. * and **: P < 0.05 and P <0.01 compared to K-Ras overexpression cells.

To verify the effect of K-Ras expression and MPE co-treatment in cell cycle progression, flow cytometry analysis was conducted. Overexpression of K-Ras significantly decreased G0/G1 phase population and increased S and G2/M phase population. Co-treatment with MPE (0.5 mg/ml) obviously enhanced G0/G1 phase population and decreased S and G2/M phase population (Fig. 4A). In addition, K-Ras attenuated the expressions of p27, p21 and p16. Nonetheless, MPE at concentrations of 0.5 and 1 mg/ml recovered the p27, p21 and p16 expressions. Although K-Ras did not affect p27 expression, p27 expression increased in the presence of MPE (Fig. 4B).

### MPE triggered K-Ras degradation

**Figure 5:**
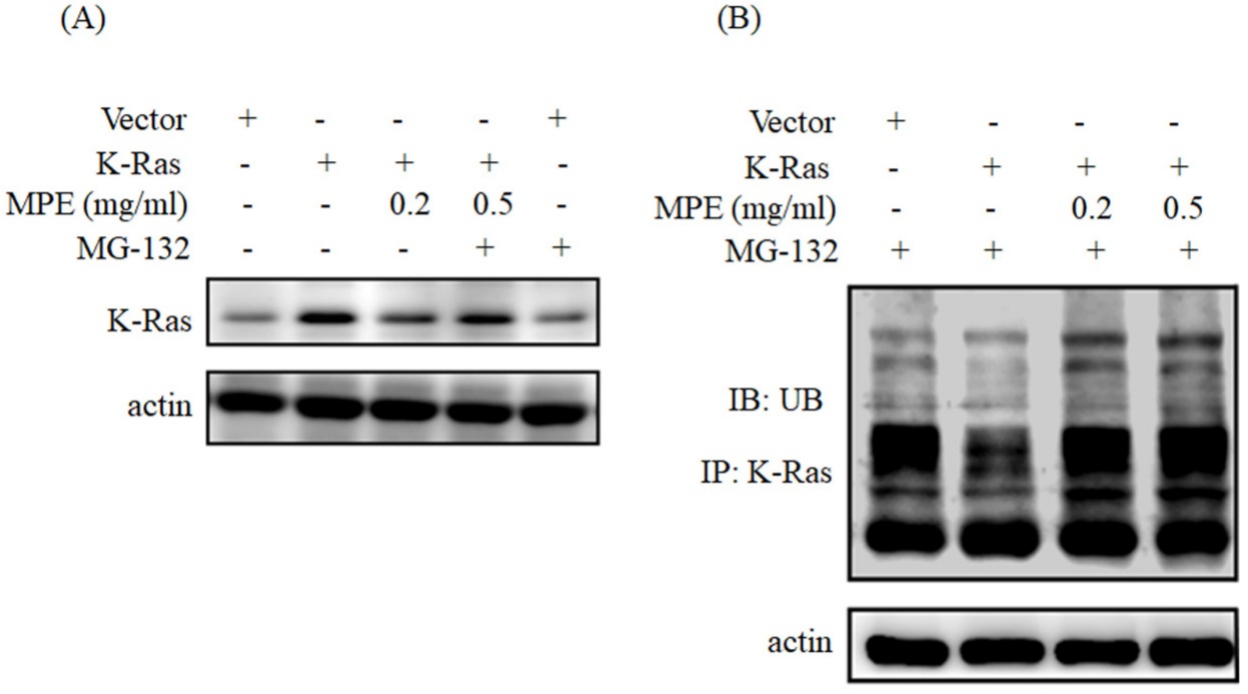
** MPE triggered the ubiquitination and degradation of K-Ras.** Proteins derived from A7r5 cells were transfected with vector plasmid. A7r5 cells overexpressing K-Ras in the presence or absence of 0.2 or 0.5 mg/ml MPE and treated with MG-132 were subjected to **(A)** Western blot analysis using anti-Ras antibody or **(B)** immunoprecipitation with anti-Ras antibody then immunoblotting with anti-ubiquitin. Actin was used as the loading control. UB denoted as ubiquitin.

To determine whether MPE triggered the degradation of K-Ras, we have pre-treated with MG132 (proteasome inhibitor) and then performed Western blot analysis. As shown in Fig. 5A, treatment with MPE (0.2 mg/ml) attenuated K-Ras expression whereas pre-treatment with MG-132 recovered K-Ras expression. Enhancement of ubiquitination was found in the presence of 0.2 or 0.5 mg/ml MPE (Fig. 5B).

## Discussion

Cardiovascular disease is among the major health burdens all over the world 18. Atherosclerosis results from hypertension, diabetes, and high fat diet and plays a critical in the development cardiovascular disease 18. Dietary flavonoids exhibit anti-inflammation, anti-oxidant, and anti-lipidemic properties and have potential as anti-atherosclerosis agents 19, 20. Herein, we provided the mechanisms underlying the action of mulberry polyphenols as anti-atherosclerosis agents through repression of proliferation and migration of Ras-overexpressing smooth muscle cells.

Damaged vascular endothelial cells attract peripheral monocyte, which differentiate into macrophage and eventually cause inflammation. Dysfunctional endothelial cells secrete growth factors, such as VEGF, basic fibrosis growth factor (bFGF), and tumor growth factor-β (TGF-β), to promote the proliferation and migration of smooth muscle cells 21, 22. Atherosclerosis can be prevented by blocking VEGF expression 22. Paeonol represses atherosclerosis by inhibiting VEGF secretion and Ras-Raf-ERK signal pathway in smooth muscle cells treated with a high amount of glucose 7. Administration of Ziziphora clinopodioides flavonoids reduced atherosclerosis formation via the attenuation of VEGF/AKT/NFkB signaling in mice fed with high-fat emulsion combined with vitamin D 3 23. We showed that the overexpression of Ras enhanced VEGF level, whereas mulberry polyphenols reversed this phenomenon and exerted anti-atherosclerosis effects.

Activation of Ras increases the activity of PI-3K and activated AKT and its downstream signals for cardiovascular disease and cancer 24. Ras triggers NFκB activation and causes NFκB translocated into the nucleus. It also regulates genes for cell migration, such as MMP-2 and MMP-9, VEGF for angiogenesis, and proteins involved in cell cycle progression for cell proliferation 25. Meng et al. demonstrated that morin hydrate represses lipopolysaccharide-induced inflammation in human umbilical vein endothelial cells (HUVECs) and ameliorates atherosclerosis formation through the inhibition of PI-3K/AKT/NFκB pathway 26. Yu et al. demonstrated that MPE blocks the migration of A7r5 smooth muscle cells through the suppression of NFkB pathway and PI-3K/AKT signals 15. Previous report indicated that MPE repressed MMP-2 activity and VEGF expression in a time-dependent manner in A7r5 smooth muscle cells 15. In the present study, the overexpression of K-Ras obviously increased PI-3K/AKT/NFκB pathway, elevated the level of VEGF and MMP-2, and eventually stimulated the migration of smooth muscle cells. Treatment with MPE reversed the K-Ras-induced phenomenon. In contrast, MPE mitigates K-Ras-induced senescence and promoted proliferation of smooth muscle cells through the downregulation of K-Ras expression and its downstream signals 17. The difference findings between this study and previous report 17 may result from distinct Ras expression pattern. Minamino et al demonstrated that transient overexpression of Ras stimulated proliferation of smooth muscle cells whereas senescence was observed after post-transfection for 72 h 27. Higher senescence and activation of Ras downstream target was found in human atherosclerosis lesions which suggested that overexpression of Ras involved in atherosclerosis 27. Moreover, MPE also caused the degradation of K-Ras via ubiquitination. Collectively, our results indicated that MPE attenuated K-Ras protein level and then blocked K-Ras target signals to prevent the proliferation and migration of smooth muscle cell.

Actin reorganization is also involved in cell migration. The Rho family proteins regulate the actin cytoskeleton. RhoA facilitates actin polymerization, whereas Rac1 and CDC42 are involved in lamellipodia and filopodia, respectively. Reports have shown that RhoA, Rac1, and CDC42 play important roles in atherosclerosis 28. Treatment with Rac1 inhibitor elevates endothelial function and reduces atherosclerosis in ApoE knockout mice 29. Chung et al. demonstrated that Nelumbo nucifera leaf polyphenol extract and gallic acid mitigate smooth muscle cell migration and proliferation through the repression of Ras and Rho A expression by miR-21, miR-143, and miR145 30. On the other hand, expression of RhoB was decreased in K-Ras overexpressed cells and recovered by MPE treatment. In line with our findings, Sun et al. indicated that miR-19a attenuates RhoB expression and then promotes migration and proliferation of smooth muscle cells 31. We showed that MPE obviously repressed RhoA and Rac1 expression and decreased phosphorylated FAK expression. Moreover, MPE reversed RhoB expression in the presence of K-Ras. Our results revealed that MPE regulated Rho family protein to prevent atherosclerosis.

Increasing proliferation by promoting the cell cycle progression of smooth muscle cells plays a pivotal role in atherosclerosis formation. K-Ras triggers cell cycle entry into the S phase and decreases the G0/G1 population. In addition, K-Ras attenuated the expressions of G0/G1 checkpoint proteins, such as p16, p21, and p27. Treatment with MPE significantly reversed the G0/G1 population and increased checkpoint proteins' expression in the presence of K-Ras. A previous report indicated that mulberry leaf extracts enhanced p53 and p27 expressions, downregulated cyclin-dependent protein kinase activities, promoted G0/G1 phase arrest, and eventually mitigated smooth muscle cell proliferation 12. Kesavan et al. demonstrated that Gentiana lutea root extracts blocked platelet-derived growth factor (PDGF)-induced smooth muscle proliferation by increasing G0/G1 phase population 32. Previous studies demonstrated that major compounds of MPE exert anti-atherosclerosis effects. Epicatechin gallate (ECG) elevates G0/G1 and reduces S phase population in smooth muscle cells in response to ox-LDL treatment and prevents atherosclerosis formation 33. Rutin inhibits the activities of mitogen-activated protein kinase (MAPK) and PI-3K/AKT pathway to mitigate the proliferation and migration of primary rat smooth muscle cells in response to glucose treatment 34. Lin et al. demonstrated that treatment with protocatechuic acid increases p53 and p21 and blocks oleic acid-induced cell proliferation of smooth muscle cells 35. Administration of caffeic acid (CA) significantly reduces oxidative stress, LDL, and triglycerides level in rats fed with atherogenic diet 33. Moreover, CA attenuates the aorta lesion region in rats fed with atherosclerogenic diet compared with those fed with normal diet 36. Zhao et al. showed that naringenin reduces the atherosclerotic plaque area through the promotion of autophagy in ApoE-/- mice fed with high fat diet 37.

Our results revealed that MPE exerts anti-atherosclerosis effects via the downregulation of the cell proliferation of smooth muscle cells.

## Conclusion

**Figure 6:**
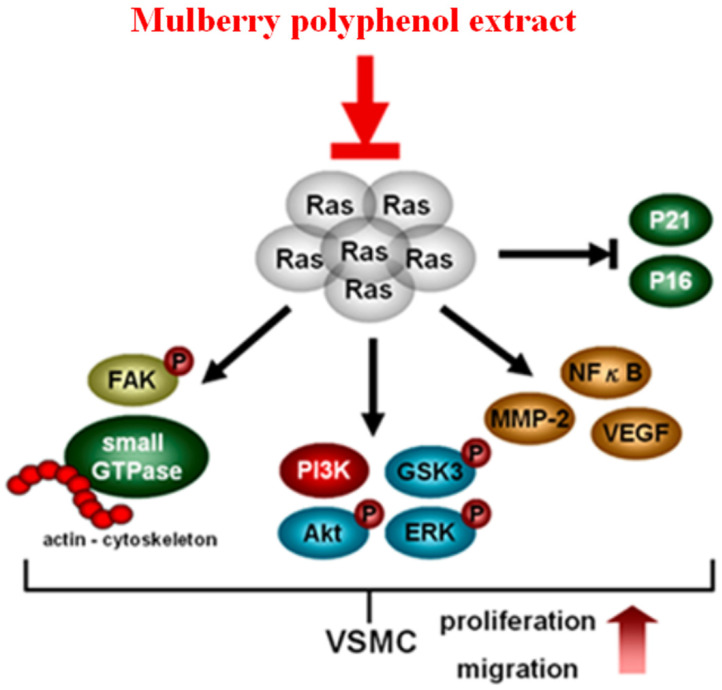
In summary, MPE triggered K-Ras degradation, suppressed Rho family proteins and FAK activities, reduced MMP-2 and VEGF expression by decreasing NFkB activity, and eventually mitigated the migration of K-Ras expression cells. MPE elevated the expressions of p27, p21, and p16 and inhibited AKT activity to repress the proliferation of K-Ras-expressing cells. Our data revealed that MPE ameliorated atherosclerosis through the regulation of the K-Ras pathway.

MPE repressed the migration of K-Ras-overexpressed smooth muscle cells by attenuating MMP-2 activity. The re-organization of actin filaments was also blocked by MPE through the downregulation of Rho A, Rac1, CDC42, and phosphorylated FAK expressions. The repression of PI-3K/AKT/GSK/NFkB signal pathway was found in MPE treatment groups. In addition, MPE elevated the expressions of cell cycle checkpoint protein, such as p27, p21, and p16, which in turn diminished cell proliferation in smooth muscle cells with K-Ras overexpression. MPE triggered K-Ras degradation, blocked K-Ras-induced smooth muscle migration and proliferation, and finally prevented atherosclerosis (Figure 6). Our results suggested that MPE has potential as an anti-atherosclerosis agent.

## Funding

This study was supported by Chung Shan Medical University and Changhua Cristian Hospital No. CSMU-CCH-110-03.and Chung Shan Medical University Hospital CSH-2022-C-018.

## Figures and Tables

**Table 1:** The polyphenolic compounds of MPE

Polyphenolic compounds	Concentration (μg/mg)
Gallic acid	2.7 ± 0.3
Protocatedchuic acid	13.8 ± 1.7
Catechin	3.2 ± 0.4
Epigallocatechin Gallate	26.3 ± 0.8
Caffeic acid	6.2 ± 1.9
Epicatechin	4.7 ± 0.9
P-caumaric acid	2.5 ± 0.2
Rutin	18.2 ± 1.1
Ferulic acid	1.0 ± 0.3
Gosspin	1.0 ± 0.1
Hersperetin	2.1 ± 0.2
Resveratrol	0.9 ± 0.2
Quercetin	6.0 ± 1.1
Naringenin	6.7 ± 1.2
Hydroxyflavin	1.4 ± 0.6

**Table 2:** The specific primers for distinct genes

Genes	Sequence	size
Ras	Sense: 5'- CTTGATAATCTTGTGTGGAAC-3'	381bp
	Antisense: 5'- CCTCCCTTTACAAATTGTAC-3'	
MMP-2	Sense: 5'-ACACCCAGTACTCATTCCCTG -3'	464bp
	Antisense: 5'-GTCCTGACCAAGGATATAGCC-3'	
VEGF	Sense: 5'-TGCACCCACGACAGAAGGGGA-3'	475bp
	Antisense: 5'-TCACCGCCTTGGCTTGTCACA-3'	
GAPDH	Sense: 5'-ACCACAGTCCATGCCATCAC-3'	451bp
	Antisense: 5'-TCCACCACCCTGTTGCTGTA-3'	
